# Ethical dilemmas surrounding patients´ “unwise” treatment preferences and suboptimal decision quality: case series of three renal cell carcinoma patients who developed local recurrences after non-guideline-concordant care choices

**DOI:** 10.11604/pamj.2024.49.45.42047

**Published:** 2024-10-18

**Authors:** Khalid Al Rumaihi, Nagy Younes, Ibrahim Adnan Khalil, Alaeddin Badawi, Ali Barah, Walid El Ansari

**Affiliations:** 1 Department of Urology, Hamad Medical Corporation, Doha, Qatar,; 2 College of Medicine, Qatar University, Doha, Qatar,; 3 Weill Cornell Medicine-Qatar, Doha, Qatar,; 4 Department of Radiology, Hamad Medical Corporation, Doha, Qatar,; 5 Department of Surgery, Hamad Medical Corporation, Doha, Qatar

**Keywords:** Cryoablation, guideline-concordant therapy, patient-centered medicine, partial nephrectomy, renal cell carcinoma, shared decision-making

## Abstract

Patient engagement and shared decision-making (SDM) between patients and clinicians is the foundation of patient-centered care. It aims to reach a treatment option that fits the patient's preference and is guideline-concordant. We sought to evaluate the possible causes and outcomes of patient's non-guideline-concordant care choices. Using a retrospective analysis of the medical records of patients who underwent cryoablation for small renal masses between January 2010 and January 2023. Inclusion criteria were patients with renal tumor(s) who underwent cryoablation which was not recommended by the multidisciplinary team (MDT). We present three patients with unilateral clear cell renal cell carcinoma. Based on imaging and other findings, the oncology MDT recommended partial/radical nephrectomy. Upon consultation, each refused surgery and preferred cryoablation. Respecting their choice, cryoablation was undertaken. The patients had treatment failure and developed recurrences that could have possibly been avoided with guideline-concordant care. Shared decision-making in healthcare involves several aspects: patient/family; uncertainty of available evidence of various treatments; MDT meetings; and treatment team. For patients to select 'wise' treatment preferences i.e. guideline-concordant care, multi-layered complex intellectual and cognitive processes are required, where experience may play a role. Healthcare professionals require guidance and training on appropriate SDM in clinical settings, and awareness of tools to solicit patient choice to guideline-concordant care whilst observing patient autonomy. Patients and treatment teams need the capacity, knowledge, and skills to reach a 'wise' guideline-concordant care treatment preference jointly. Patients' unwise preference could lead to suboptimal outcomes, in the case of our patients, tumor recurrence.

## Introduction

Treatment decision-making processes pass through three stages: information exchange, deliberation, and deciding on the treatment to implement [1]. Effective patient-healthcare provider communication promotes the awareness and appreciation of patients to their illnesses, available treatment options, and adherence, which is essential for managing the condition [2]. Patient engagement (PE) and shared decision-making (SDM) between the patient and clinician (s) is the foundation of patient-centered care, premised on a respectful dialog where the patient's preferences and knowledge of the physician interact to generate an optimal decision [3]. Hence in SDM, healthcare professionals (HCP) and their patients together have to decide upon the treatment option that best fits the patient's situation and preference [4]. By improving their relationships with patients, HCPs can enhance SDM [5].

A body of literature suggests that such notions are associated with better outcomes. For instance, for the treatment of depression, PE in decision-making was associated with the receipt of more adequate treatment, guideline-concordant care, and satisfaction [6,7]. Patient preferences are viewed favorably, to the extent that recent mental health law reforms in the UK prioritize patient choice [8]; and in the USA, approaches to prenatal care delivery incorporate patient preferences [9].

But patients might not be prepared to engage in SDM, or desire to be fully involved [3]. Patients with carpal tunnel syndrome had varying degrees of involvement in their care decision-making, preferring a semi-passive role in their intra/postoperative decisions [10]; and for vascular diseases, SDM remains low [4]. In addition, patients might be at risk of suboptimal decision quality: breast cancer care is characterized by preference-sensitive decisions where no single choice dominates, and the management approach is guided by patient values/preferences, but due to the complex choices, patients might be vulnerable to suboptimal decision quality, such as the timing of adjuvant radiation with regards to its toxicity, cosmetic effect, and oncological outcomes [11].

In such cases, the perceptions of patients and clinicians might not be congruent, raising the question of what happens when the patient's and physician´s views do not align [12]. For gynecological cancer care, the perspectives of patients and clinicians aligned on many topics but diverged on others [12] If one assumes that the practitioner is right (a subject of research in itself), then how do we get the patient to follow that advice [13]? A high-quality, patient-centered decision involves an accurate understanding of the risks and benefits of treatment options, as well as concordance with the patient's preferences [11].

As for the treatment of clinically localized renal masses, the four major management strategies available comprise radical nephrectomy, partial nephrectomy (PN), cryoablation (CA), and active surveillance. Each management strategy is associated with a unique profile of functional and oncological outcomes. The choice of the management modality that suits the patient depends on tumor characteristics such as size, complexity, and nature (whether cystic/solid), along with patient factors such as age, comorbidities, and renal function [14]. The multiple treatment options and factors that influence the choice of the best treatment modality highlight the importance of an oncology multidisciplinary team (MDT) in such cases.

We present a retrospective analysis of three non-consecutive patients diagnosed with unilateral clear cell renal cell carcinoma (RCC). For each patient, based on imaging and other findings, the oncology MDT recommended partial/radical nephrectomy. However, upon consultation and counseling with the patients, each refused surgery and preferred cryoablation. Although healthcare in Qatar is universal and free of charge, eliminating financial factors that might direct patients toward lower-cost treatment modalities, cryoablation was undertaken in keeping with the patient's choice of treatment. However, the three patients subsequently developed recurrences. The specific objectives are to: a) describe and outline the cases; b) highlight that the cases chose non-guideline concordant care; c) explore the possible reasons why the cases made such choices; d) discuss the processes, discourses, and implications of such choices; and, e) emphasize some useful available tools that healthcare professionals can use to assist patients in making guideline-concordant treatment choices.

## Methods

**Study design:** a retrospective analysis of the medical records.

**Setting:** Department of Urology, Uro-Oncology Unit, Hamad Medical Corporation in Doha, State of Qatar.

**Cases:** three non-consecutive patients who underwent CA for small renal masses during the period January 2010 to January 2023 at our institution and subsequently developed recurrence.

**Variables:** we retrieved demographic (age, sex, nationality, medical history, comorbidities, socio-economic status, education, occupation), radiologic (imaging modality, date done, laterality, tumor size, tumor nature, nephrometry score, tumor stage), MDT recommendation) and other relevant cryoablation (date, number of needles used, gas used, number of freezing cycles, biopsy results, tumor grade) data.

**Data sources/measurement:** we used hospital electronic databases and medical imaging databases. In addition, we also gathered from the databases any available socio-demographic data and/or medical history information that could aid in explaining the potential reasons behind the non-guideline-concordant care choices that these patients selected, as well as assist in the patients´ judgments and processes for refusing surgery. These variables included age at procedure, sex, nationality, medical history and comorbidities, socioeconomic status, education, and occupation.

**Inclusion/exclusion criteria:** the inclusion criteria were all patients with renal tumor (s) who selected and underwent CA despite that it was not recommended by the MDT. Cases that did not fulfill the inclusion criteria were excluded.

**Statistical methods:** we sought to qualitatively evaluate the possible causes and outcomes of non-guideline-concordant care choices. Hence, no statistical analysis was required or undertaken.

**Counselling and patient choice at our institution:** during the counseling, any MDT decision was thoroughly discussed with each patient in his/her native language or with an interpreter where required, highlighting that the recommended intervention is beneficial for the patient's health. In addition, patients were provided with a set of useful educational electronic internet links. The details of the proposed surgical treatment with its expected outcomes, benefits, and possible complication (s) were meticulously discussed and reviewed with the patient. Other available alternative treatments (e.g. ablative treatments, active surveillance) [14] were also deliberated with the patients, again each with its excepted outcomes, benefits, and possible complication (s) and their probabilities. For instance, counseling patients about PN versus CA in the management of renal masses includes clarifying the higher risk of local recurrence and lower oncological outcomes of CA compared to PN. However, CA is associated with lower perioperative complications and better preservation of renal function. Patients are then given one week to think and reflect on the information provided and receive a second opinion if patients wish to do so. Patients were encouraged to return if they had any queries, concerns, or uncertainties; and to arrange and schedule the treatment modality that they select.

**Multidisciplinary team membership:** consisted of senior consultants in uro-oncology, medical oncology, radiology, interventional radiology, and histopathology.

**Cryoblation technique:** the procedure is performed in the prone position under general anesthesia using an angio-CT suite. A planning triphasic CT scan is performed to outline the lesion, and a core biopsy is taken from the renal tumors using a coaxial system 18G x 16cm biopsy needle at the beginning of the procedure before the start of CA. Additionally, a coil marker is placed into the lesion through the already placed co-axial needle. A 16G CA needles are placed into the renal lesion the number of needles is changed according to the tumor size to make sure that the whole renal lesion is included in the CA ball. Cryoablation using Argon gas is started for 10 minutes followed by passive thawing until the temperature reaches 0° Celsius which is followed by a 2^nd^ cycle of CA for 10 minutes followed by active thawing using Helium gas until the temperature reaches 30° Celsius and then the needles are removed.

**Follow-up post cryoablation:** periodic medical history, physical examination, laboratory studies, and pre-and post-contrast abdominal imaging within 6 months (if not contraindicated) [14], subsequent follow-up is scheduled according to the MDT recommendations.

**Ethical consideration:** informed consent was obtained from the subjects involved in the study.

**Patient engagement (PE):** the desire and capability to actively choose to participate in care in a way uniquely appropriate to the individual, in cooperation with a healthcare provider or institution, to maximize outcomes or improve the experiences of care [15].

**Shared decision-making (SDM):** an approach where clinicians and patients share the best available evidence when faced with the task of making decisions, and where patients are supported to consider options, to achieve informed preferences [16].

**Optimal treatment strategies:** the decision rule that leads to the most beneficial outcome on average or the greatest value [17].

## Results

We found a total of 3 cases with renal tumors who preferred CA treatment despite that MDT recommended partial/radical nephrectomy.

**Figure 1:**
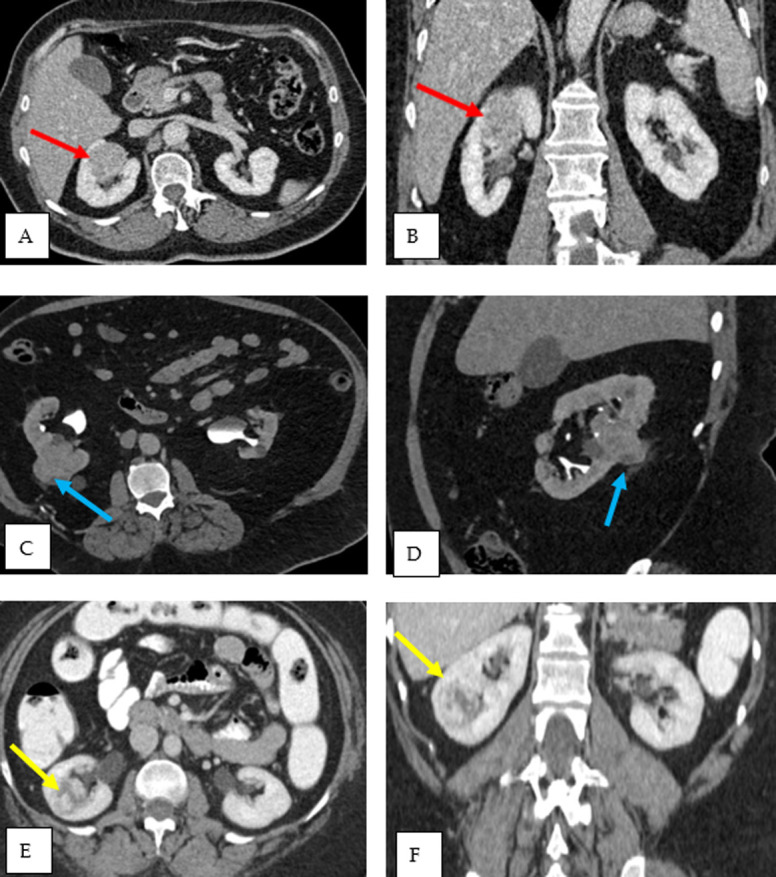
renal masses of three patients by contrasted CT scan; patient 1 (A, B) central right renal upper to mid pole solid lesion (4 x 3.5 x 3.5 cm, red arrows); patient 2 (C, D) heterogeneous enhancing exophytic mass lesion arising from the mid pole posterior cortex of right kidney, protruding into right middle lobe calyx (5.4 x 4.8 x 4 cm, blue arrows); patient 3 (E, F): completely endophytic heterogeneous enhancing lesion arising from lower pole of right kidney (2.5 x 3 x 3 cm, yellow arrows)

**Patient socio-demographics and diagnosis:** patients´ age ranged between 56 to 68 years and the sample comprised two males and one female. All three patients underwent percutaneous CA as described above. Table 1 lists the particulars of the patients´ socio-demographics, MDT plan, and original tumor characteristics. Figure 1 depicts the investigations and diagnosis of the initial renal masses of the three patients.

**Table 1:** patient demographics, MDT plan, original tumor characteristics, and cryoablation details

Characteristic	Patient 1	Patient 2	Patient 3
**Socio-demography**			
Age at procedure (years)	68	64	56
Sex	Male	Male	Female
Nationality	Jordanian	Lebanese	Qatari
Medical history and Comorbidities	DM, HTN, Colon CA	DM, CAD	HTN
Socioeconomic status	Middle	Working	Upper
Education	Bachelor’s degree	High school	PhD
Occupation	School teacher	Car mechanic	University faculty
**Diagnosis**			
Imaging modality	CT abd with contrast	CT abd with contrast	CT abd with contrast
Date	March 2017	February 2021	November 2017
Laterality	Right	Right	Right
Tumor size (cm)	4.0 * 3.5 * 3.5	5.4 * 4.8 * 4	2.5 * 3 * 3
Tumor nature	Solid tumor	Heterogeneous (mixed cystic and solid)	Heterogeneous (mixed cystic and solid)
Nephrometry score	7	8	6
Tumor stage	cT1b	cT3a	cT1a
MDT recommendation	Partial nephrectomy	Radical nephrectomy	Radical nephrectomy
**Cryoablation**			
Date	May 2017	April 2021	January 2018
Details			
Number of needles used	**—**	3	2
Gas used	**—**	Argon	**—**
Number of freezing cycles	**—**	2	2
Biopsy results	**—**	Clear cell RCC	Clear cell RCC
Tumor grade	**—**	2	1


DM: type 2 diabetes mellitus; HTN: hypertension; CA: cancer; CAD: coronary artery disease; CT: computed tomography; MDT: multi-disciplinary team; abd: abdomen; —: data not available

**Figure 2:**
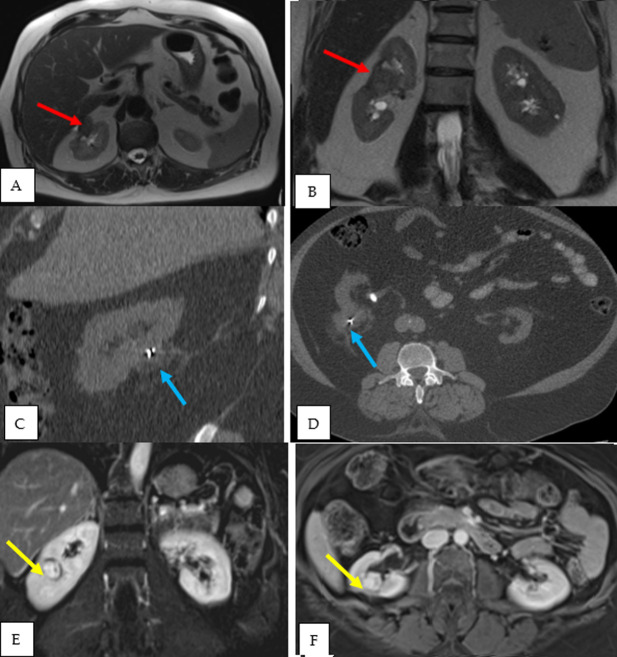
recurrence of renal masses of three patients posts cryoablation; patient 1 (A, B) MRI with recurrence at tumor bed (27 x 18 mm, red arrows); patient 2 (C, D) CT scan showing slightly cystic hypoechoic focus posterior to right kidney, suggestive of tumor recurrence (3.6 x 2.9 cm, blue arrows); patient 3 (E, F) MRI showing focal heterogeneous lesion abutting the renal sinus fat, suggestive of tumor recurrence (18 mm, yellow arrows)

**Tumor recurrence:** tumor recurrence was detected on follow-up imaging. The mean duration for tumor recurrence was 30.6 months. Recurrence was managed by redo-cryoablation in patient 2 and patient 3, while patient 1 omitted management of recurrence as he was diagnosed with metastatic colon cancer. The characteristics of tumor recurrence are listed in Table 2 and depicted in Figure 2.

**Table 2:** tumor recurrence characteristics

Characteristic	Patient 1	Patient 2	Patient 3
Recurrence			
Imaging modality	MRI Abd with contrast	MRI Abd with contrast	MRI abd with contrast
Date	July 2019	March 2022	August 2020
Duration to recurrence (month)	26	11	55
Size (cm)	2.7 * 1.8	3.6 * 2.9	1.8 * 1.2
Management	None	Redo cryoablation	Redo cryoablation

MRI: Magnetic resonance imaging; abd: abdomen

## Discussion

### "No decision about me, without me"

With person-centered care, patients take substantial roles in decision-making [18,19]. Patient engagement is essential for quality care and patient safety [18] and works by transforming new knowledge and values into behaviors [20]. The current report provides insights into the complex ways patients make decisions and engage in selecting their treatments. We presented three clear cell RCC cases where patients practiced their preference and unfortunately selected non-guideline-concordant care, contrary to their HCPs' recommendations, with treatment failure and recurrences that could have been avoided should they have selected guideline-concordant care.

Shared decision-making comprises dialogue, where patient's preferences interact with the physician's knowledge to reach optimal decisions [3]. Shared decision-making in healthcare involves four aspects: the patient (and family); prevailing uncertainty of the available evidence for various treatment options; the dynamics of MDT meeting (s); and the treatment team. Patients need to have the capacity to process complex information and treatment options and make decisions in their best interest. Healthcare professionals need to have the capacity to lay out the necessary information to assist patients in making 'wise' choices, and the capability to advise them when they select non-guideline-concordant care that could result in suboptimal outcomes. Uncertainty of the evidence adds more complexity as potential outcomes of treatment options are expressed in probabilities and likelihoods.

**Potential reasons for refusing surgery and preferring cryoablation:** the MDT decisions were PN (patient 1) and radical nephrectomy (patient 2 and patient 3). The outcomes and possible complications were clarified to the patients. In Qatar, healthcare is free, hence the financial burden of the treatment options is not a determining factor of the selected choice (no out-of-pocket expenses). The patients refused surgery due to possible complications (bleeding, cosmetic effects of scars, visceral injury, renal function deterioration, etc.) [14]. They preferred CA although it was not the MDT-recommended intervention possibly due to the tumor´s location, cystic nature, and size. Still, CA has a higher risk of treatment failure and recurrence [14,21].

### Patients and decision-making

Unwise decision-making poses ethical challenges for physicians and would reappraise a patient´s capacity if she/he presents with such a decision [22]. Ethical care builds on beneficence and nonmaleficence [23,24], and HCPs are contested when patients present with suboptimal decisions [25]. Patients´ health literacy and numeracy influence their decision preferences [26], or they might not desire to make decisions, choosing to trust the physician [27-29], particularly when physicians provide reasoning [30-33], entrusting them with final decisions [34].

Regarding patient capacity, Table 1 shows that patients 1 and 3 were of middle/upper socioeconomic status, well-educated, and employed in intellectual jobs. This suggests that, provided with information about treatment options and outcomes, both patients could make optimal decisions. It is difficult to speculate the reasons behind a choice that a patient makes, however, patient 1 had a history of several surgical procedures: rectal cancer (2008) resected but complicated by incisional hernia, tumor recurrence, and intestinal obstruction; exploration laparotomy, ileostomy, hernia repair (2009); ileostomy revision and recurrent hernia repair (2011); then right renal mass and offered partial nephrectomy (2017). Such a surgical history might have influenced his decision-making away from the surgical option to ablation despite its lower success rate [35,36].

On the other hand, patient 3, being female, might have preferred ablation for aesthetic reasons (lack of visible surgical scar). Conversely, case 2 was a car mechanic with a high school education, and no history indicators to provide clues to his non-guideline-concordant choice. He might have believed that surgery could affect his job which requires physical fitness. Our findings concur with that socio-demographic features e.g., being female and greater educational attainment, higher health literacy/numeracy, and disease severity influence the extent to which patients desire active involvement in care decisions [26,37].

A chosen preference might be explained by its benefits and negative consequences. Different options have their risks (probability of adverse outcomes) and benefits [3,4], and patient preference builds on their perceptions of these [38]. Patients value the use of personalized risks when deciding treatment [39], and their decisions relate to the perceived benefits and negative consequences [40]. Ansari's paradox suggests that for individuals to perceive favorable or very favorable cost-benefit ratios, their perceptions of benefits needed to be 62%-82% more than the negative consequences [40]. Social exchange is the actions of individuals motivated by the expected returns [41]. Hence, our patients were more likely to choose guideline-concordant care if they perceived its benefits to be considerably more than its negative consequences. However, the benefits and negative consequences of treatments are premised on complex estimations, interactions between clinical evidence of variable quality, and patients´ appreciation of such evidence. Although uncertainty should be disclosed, in cancer treatment decision-making, uncertainty was disclosed in only 34% of consultations [39].

### Available evidence, probabilities, and certainty

For our patients, evidence suggests the postoperative complications were more for PN vs CA (42% vs 23%), albeit CA has higher local recurrence [21]; and compared to PN, CA for cT1 renal tumors yields inferior results [36]. There seems no consensus on the criteria to select the best patients, although due to the higher rates of CA treatment failure, it is seldom offered to patients with less comorbidities and good life expectancy [42]. Ablation may have worse local recurrence and metastasis outcomes [35], with increased mortality among patients with pT1b RCC [43]. Patients should be counseled on such increased odds of tumor persistence or local recurrence after ablation compared to surgery [14].

The main challenge for care teams is that it is impossible to point out the patient who would benefit. Our three cases selected ablation, and despite being non-guideline concordant, there was no absolute certainty that any of these patients would develop a recurrence, only a likelihood. Hence, preventing a recurrence by selecting a guideline-concordant option is not guaranteed. Such a “prevention paradox” highlights that prevention strategies offering large health benefits might realize fewer benefits at an “individual” level. A preventive measure that brings large benefits to the community may offer little to most participating persons [44]. There is no single “correct” or “best” management plan; rather, more or less “reasonable” or “defensible” plans [45].

### Multidisciplinary team meetings

Multidisciplinary team meetings represent the backbone of clinical management [46]. Our patients were discussed at the MDT meetings; hence they were more likely to receive more accurate and complete pre-operative staging and better accordance with clinical guidelines for treatment, thus resulting in better outcomes [47,48]. However, cancer care is continuously challenged due to contemporary management regimes, multi-modal therapies, and survivorship issues [46]. Traditionally, patients are not physically present at MDT meetings, and there have been calls that patients be actively integrated into MDT processes to ascertain they have informed choices and ensure that recommendations are premised on the best available evidence [34].

Multidisciplinary team meetings are most beneficial when patient choice prevails, but patients are usually seen after the meeting [49]. Barriers to the implementation of MDT suggestions encompass not considering patient choices [50]. Bladder and prostate cancer guidelines [51,52] do not recommend using synchronous joint clinics, despite that most patients seen in synchronous joint uro-oncology clinics preferred joint consultations [49]. Given the information complexity, HCPs' roles necessitate skills and tools for patient-centered communication and visual displays [53]. Shared decision-making can be enhanced by HCPs skillfully aiding their patients to debate their options [4].

**Physicians and skill sets:** from the physicians' side, the patient-centered approach respects patient preferences to steer clinical decisions [54]. However, HCPs have little guidance on how to accomplish this [55]; and clinicians might be poor at judging patients´ treatment preferences [56]. Patients' preferences require clear information from HCPs [57,58], and HCPs need to appraise whether the patient understood the options, e.g., by asking the patient to repeat or paraphrase the options [59]. Explanations to patients of the links between a selected preference and resultant outcomes help them make informed decisions, and we undertook this with our patients. Skills for effective SDM are not taught in medical schools. In Holland, SDM was low due to unsatisfactory patient support to debate the options, and training on SDM consultations was required [4]. Interventions to enhance patient involvement in treatment result in increased use of services, more patients receiving their preferred treatment, and better outcomes [60-66]. However, a thin line exists between aiming to modify patients´ desires and beliefs, and intrusively affecting PE which endangers patients´ autonomy [67].

### Tools and techniques for a patient-centered approach

These include patient-centered communication, patient education and counseling, management reasoning, and nudging. Patient-centered communication predicted the patient´s continuous adherence 36 months after diagnosis [68]. Management reasoning is premised on negotiating a plan, with ongoing monitoring and modifications of the plan. These necessitate communication abilities, negotiations with patients, and appraisals of the reasoning processes [45]. Justifying the rationale for given management plans commands that clinicians are effective communicators [69], and the race/sex of the patient and surgeon could influence perceptions of such communication [70]. Healthcare professionals might not hold the skills necessary for counseling [71] or lack the confidence to effectively communicate with patients [72]. Improved HCP communication in providing patient counseling reduces the risk of adverse medication problems and readmissions [73]. Management reasoning is whereby clinicians combine clinical data medical knowledge, and patient preferences to suggest management decisions for individual patients [45]. Nudge acts by modifying the architecture of choice, using techniques to encourage people to modify their behavior by employing gentleness rather than coercion [74]. Healthcare professionals need to furnish patients with clear and detailed numerical risk information and clarify how personalized side-effect risks are assessed [39]. The extent of knowledge of and use of such techniques by the treatment teams with our patients is not entirely clear.

This study has limitations. Retrospective interrogation of data has its inherent limitations. It would have been beneficial to interview our cases and receive their perspectives on their decision-making. For future research, we agree with others [75], about the limited appraisals of the reasons why and how patients decide between various treatments, and assessments of patients' views before and after a given choice. Future research should also examine the impact of potential risks on quality of life compared to the oncological outcomes of treatment modalities to undercover patients´ treatment choices and processes underlying treatment decisions.

## Conclusion

Selection of guideline-concordant care treatment preferences by patients involves multi-layered complex intellectual and cognitive processes. Patients and the treatment teams need to have the capacity and requisite knowledge and skills to reach a 'wise' guideline-concordant care treatment preference jointly. Patients´ unwise treatment preferences could lead to suboptimal outcomes, in the case of our patients, tumor recurrence.

### What is known about this topic

- Ethical dilemmas in patient treatment preferences highlight the pivotal role of patient autonomy in healthcare decision-making;
- Ethical challenges emerge in effectively communicating the risks and outcomes of non-guideline-concordant care choices, requiring clinicians to maintain truthfulness while respecting patient autonomy;
- The role of shared decision-making has not yet been described in the management of renal cell carcinoma (RCC), where there are rapidly developing treatment options and an expanding evidence base.

### What this study adds

- Comprehensive understanding of the process of shared decision-making for renal cell carcinoma and the effect on patient adherence to the recommended plans and guideline-concordant care choices;
- Detailed description of the available tools that healthcare professionals can use to assist patients in making 'wise' treatment choices, and avoiding 'unwise' choices;
- A deeper understanding of the factors at play in patient decision-making, advocating for adherence to established guidelines to maximize treatment effectiveness and patient well-being; it underscores the significance of collaborative decision-making between patients and their treatment teams, highlighting the need for both parties to possess the necessary knowledge, skills, and abilities for informed choices that are aligned with guidelines.
